# Identifying the key biophysical drivers, connectivity outcomes, and metapopulation consequences of larval dispersal in the sea

**DOI:** 10.1186/s40462-015-0045-6

**Published:** 2015-07-15

**Authors:** Eric A. Treml, John R. Ford, Kerry P. Black, Stephen E. Swearer

**Affiliations:** School of BioSciences, University of Melbourne, Parkville, Victoria, 3010 Australia

**Keywords:** Dispersal, Life history, Local retention, Self-recruitment, Sensitivity analysis

## Abstract

**Background:**

Population connectivity, which is essential for the persistence of benthic marine metapopulations, depends on how life history traits and the environment interact to influence larval production, dispersal and survival. Although we have made significant advances in our understanding of the spatial and temporal dynamics of these individual processes, developing an approach that integrates the entire population connectivity process from reproduction, through dispersal, and to the recruitment of individuals has been difficult.

We present a population connectivity modelling framework and diagnostic approach for quantifying the impact of i) life histories, ii) demographics, iii) larval dispersal, and iv) the physical seascape, on the structure of connectivity and metapopulation dynamics. We illustrate this approach using the subtidal rocky reef ecosystem of Port Phillip Bay, were we provide a broadly-applicable framework of population connectivity and quantitative methodology for evaluating the relative importance of individual factors in determining local and system outcomes.

**Results:**

The spatial characteristics of marine population connectivity are primarily influenced by larval mortality, the duration of the pelagic larval stage, and the settlement competency characteristics, with significant variability imposed by the geographic setting and the timing of larval release. The relative influence and the direction and strength of the main effects were strongly consistent among 10 connectivity-based metrics.

**Conclusions:**

These important intrinsic factors (mortality, length of the pelagic larval stage, and the extent of the precompetency window) and the spatial and temporal variability represent key research priorities for advancing our understanding of the connectivity process and metapopulation outcomes.

**Electronic supplementary material:**

The online version of this article (doi:10.1186/s40462-015-0045-6) contains supplementary material, which is available to authorized users.

## Background

Understanding the causes and consequences of dispersal is a foundational goal in population and community ecology, and evolution. Dispersal, or the exchange of individuals among natal and non-natal sites, is the primary process that ‘connects’ populations, with important impacts on local demography, landscape-wide population dynamics and gene flow. Although there are a diversity of evolutionary drivers of dispersal, such as reducing kin competition and inbreeding [1], ‘bet hedging’ offspring success through spatial-temporal variation in reproduction [2], and escaping unfavourable or ephemeral local conditions [3], it is the more proximate drivers of dispersal at ecological time scales which influence the selective pressures on dispersal traits [4]. At ecological time scales, population connectivity is critical for population growth [5, 6] and system persistence [7], aids in the local-scale recovery or rescue from severe disturbances [8], and plays a key role in driving metapopulation dynamics [9]. Connectivity is also believed to be important in determining how species will cope in a changing climate by allowing species’ ranges to expand or constrict in response to environmental shifts [10].

The importance of population connectivity has also fuelled efforts to integrate this process into conservation and management, particularly in relation to population persistence and viability [11], reserve design and spatial management strategies [12], and the alignment between management efforts and ecological processes [13]. Unfortunately, identifying quantitative conservation objectives with respect to connectivity remains difficult, particularly in regards to the specific process, landscape feature, or population outcome being targeted for conservation [14]. For example, targeting strong source habitat patches (self-sustaining subpopulations that are net exporters of individuals) would lead to different conservation outcomes than if one were to target locally persistent patches, critical stepping-stones, or sites receiving the highest diversity of settlers [15]. Clearly, a more holistic understanding of the drivers of population connectivity and the population and system-wide implications is needed.

Recent advances in movement ecology, population biology, and land/seascape ecology have improved our understanding of this biological-physical process. The study of the mechanisms of dispersal movements *per se* has elevated the importance of both intrinsic (biological) and extrinsic (e.g., environmental) drivers [16, 17] and the role of individual-based decisions in determining dispersal outcomes [18, 19]. This work appears to be coalescing into the study of dispersal syndromes [20, 21], or the patterns of covariance between dispersal potential and suites of life history traits based on shared evolutionary or environmental histories [22]. Concurrently, the exploration of the patterns and implications of dispersal has often taken a spatially-explicit or landscape ecological approach, which has increased our ability to quantify the impact of matrix structure and habitat topology on connectivity and how it interacts with life history traits in determining broad-scale emergent patterns of metapopulation dynamics [23]. The mechanistic approaches to studying population connectivity have not been well integrated with landscape ecological approaches, largely due to the differences in spatiotemporal scales. Unifying these often disparate approaches, however, would provide a more holistic quantitative framework for investigating the factors that influence dispersal-driven connectivity and their population level consequences.

Studying dispersal-driven population connectivity in benthic marine species with complex life cycles is particularly challenging due to the strong influence of currents, the age, size, and behavioural complexities of the dispersing individuals, and the spatiotemporal scales (and variability) of the process [24]. Despite these difficulties, technological advances in larval tagging, computation power and model sophistication have enabled significant progress in estimating the scales of connectivity and identifying several key drivers for a number of taxa [25]. At local scales, the proportion of total larvae released that ultimately recruit back to the natal population, termed local larval retention [26, 27], is essential for determining demographically meaningful estimates of population replenishment [28] and quantifying a population’s dependence on subsidies from non-local sources for population persistence [29]. Local retention is driven by local-scale hydrodynamics or ‘sticky water’ [30], early-stage larval behaviour [31], and aspects of the local habitat structure [32], with estimates as high as 20 to 30 % in some systems [33, 34]. Together, local retention and the amount of immigrating larvae arriving from upstream sources determine the relative dependencies on natal and non-natal larvae to population growth and persistence. Although measuring this mixture between natal and non-natal recruits is becoming easier with genetic [35] and otolith-based [27] approaches, estimates must be viewed in the context of the local population size, reproductive output, and local demographic rates to determine relevant recruitment rates [28].

Although individual biophysical parameters are important in determining connectivity outcomes, such as larval mortality [36], larval behaviour [31] and sensing [37], reproductive output [32], duration of the pelagic stage [38], and local-scale ocean physics [39–41], we have very little understanding of the relative importance and interactions of these parameters in any given system or for any particular species (but see [40, 32]). To move beyond one-at-a-time empirical evaluations of parameter importance, we are largely dependent on models to develop a more comprehensive understanding of this complex system across scales [42].

**Fig. 1:**
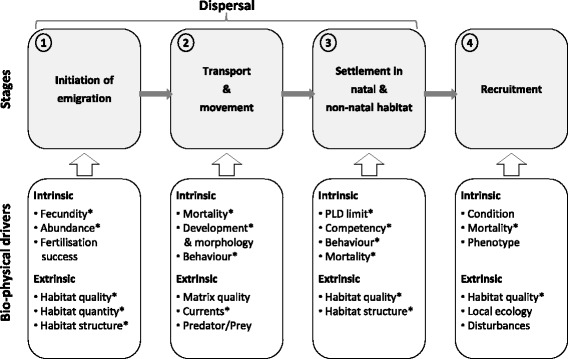
Conceptual framework of the processes and drivers of population connectivity. Population connectivity refers to the exchange of individuals resulting from their biophysical dispersal, retention, and post-settlement survival. This 4-stage process may impact local patch demographics, metapopulation dynamics, and gene flow, and is spatially and temporally context dependent. Drivers highlighted with (*) are included in the modelling example of Port Phillip Bay

Here we have taken a system-level perspective and define metapopulation connectivity as the aggregate process integrating natal dispersal, post-settlement survival and reproduction (i.e., recruitment), in both natal and non-natal sites. This process-based conceptualisation includes four-stages of population connectivity (Fig. 1) and provides a clear framework for investigating the primary intrinsic and extrinsic drivers of marine larval dispersal [43] and the local to population-wide consequences. This connectivity definition is consistent with the recent marine literature [43] and genetic descriptions [44], and incorporates the three phases of dispersal common in the movement ecology field [45, 20, 46, 18]. Guided by this framework, we have used a well-validated, high-resolution, and three-dimensional biophysical model of marine larval dispersal to gain a better understanding of the drivers of marine population connectivity and its metapopulation implications. Specifically, we have three primary aims: i) to present a process-based conceptual framework of marine population connectivity and their intrinsic and extrinsic drivers, ii) to quantify the relative importance of individual parameters in determining population connectivity outcomes, and iii) to identify important knowledge gaps and prioritise research questions to improve our understanding of population connectivity.

## Methods

### Population connectivity framework

Due to the nearly passive dispersal qualities of the early developmental stages, the strong influence and dynamics of the physical environment (i.e., currents), and the potential mobility and sensing capacity of late-stage dispersers, the existing mechanistic models of dispersal are inadequate. As a result, we have developed a framework describing the four stages of population connectivity, incorporating the three stages of dispersal [47, 20] appropriate for both vector-mediated passive dispersal [46] and active dispersers, and includes the final stage of recruitment (post-settlement survival to reproduction), which is critical in determining ‘realised’ connectivity [48] and important in evolutionary models of dispersal [49]. This framework illustrates the key intrinsic and extrinsic drivers acting upon each stage of connectivity and captures the complex bio-physical and contextual interdependencies of this process characteristic of marine and aquatic environments (Fig. 1).

The first stage in the connectivity process is the initiation of emigration (‘departure’ in [20], ‘initiation’ in [46]) in which some quantity of gametes, spores, or larvae are released from the parent. Once released, this stage is followed by the transport and movement stage [‘transience’ in 20, ‘transport’ in 46] where the disperser’s trajectory is determined both by the potential advection and turbulence of currents, and the motility and behaviour of individuals, often extending from days to weeks, with wide variability among taxa [50]. Settlement marks the end of the dispersal period [‘termination’ in 46, ‘settlement’ in 20] in which dispersers actively settle to some suitable habitat patch, either within the natal source site or in a non-natal location. Individuals successfully settling into viable habitat enter the final stage of recruitment, in which some may survive and mature to reproduce, thereby contributing to subpopulation demographics and gene flow. Together, these four stages of population connectivity represent the unique biophysical processes determining the connectivity of subpopulations.

The parameters important in emigration are related to reproductive output [or vector seed load in 46], and include the fecundity, abundance, and fertilisation success of the parents [51]. Reproductive output is strongly context-dependent and influenced by extrinsic drivers such as the quality, quantity, and spatial structure of the natal habitat, and their effect on individual parents [phenotype-dependence, 20]. The transport and movement stage depends on mortality [52], larval development [51], individual sensing and motility [37], and the extrinsic role of currents [40] and spatial habitat structure [32]. Settlement can be as biophysically complex and governed by intrinsic (settlement competency window, behaviour, motility, and sensing) and extrinsic factors (habitat quality and structure and currents) and is likely context- (phenotype-environment mismatch, [53]) and condition-dependent [54, 55]. Similarly, once settled, recruitment into the adult stage is determined by individual growth and survival to maturation, which are influenced by habitat quality, competition, and individual condition. This conceptual model of population connectivity effectively partitions the primary intrinsic and extrinsic parameters among the key life stages enabling the relative importance of each parameter and stage to be quantified.

### Test case: bay-wide marine population connectivity

**Fig. 2:**
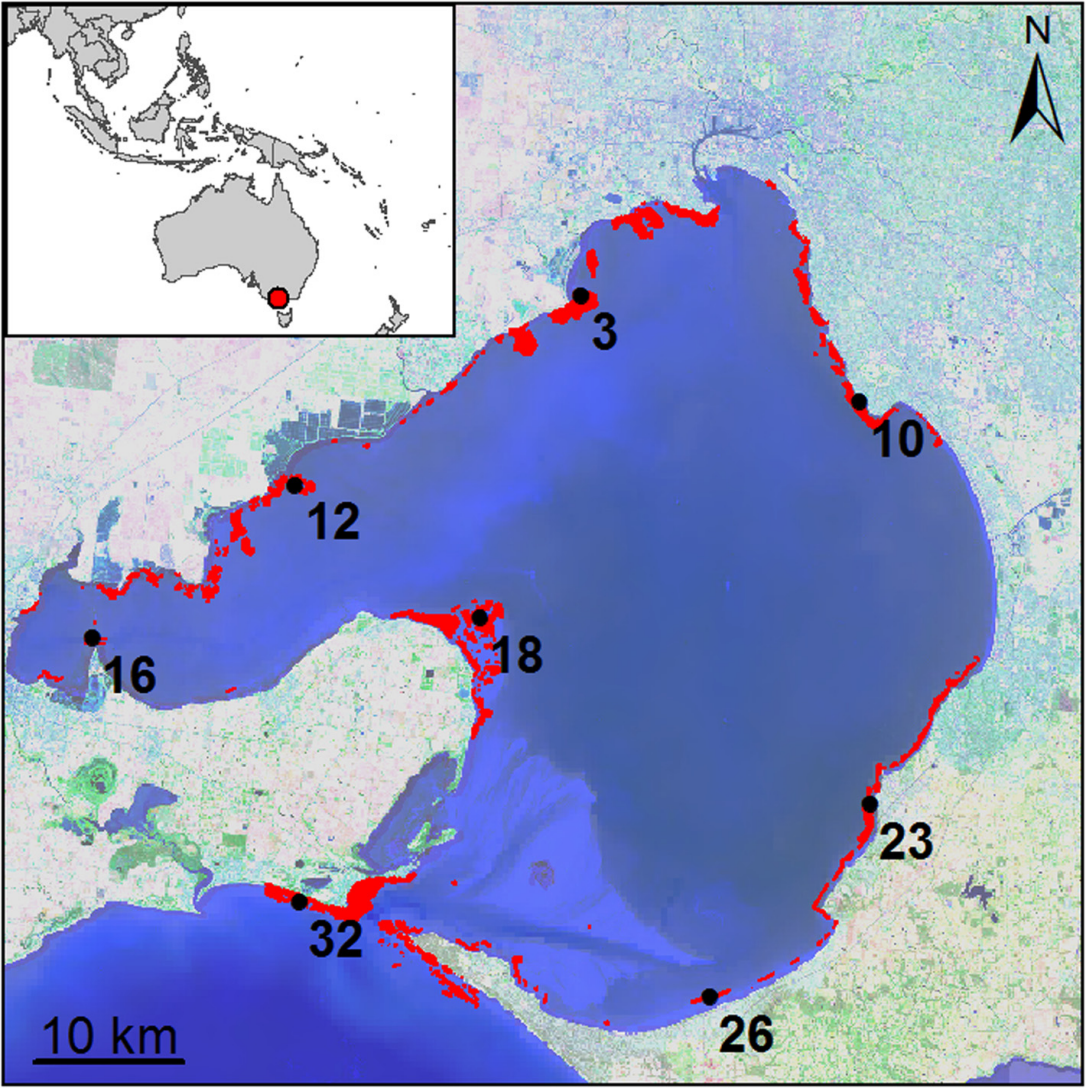
Study area of Port Phillip Bay, Victoria, Australia, used in the case study. Rocky reefs are highlighted in red, with the eight patches used in the analysis labelled. Map in a Mercator projection

Port Phillip Bay (PPB) is a large (~2,000 km^2^) semi-enclosed temperate marine system in Victoria, Australia, and is ideally suited for exploring the biophysics of marine population connectivity. One of the most prominent and economically important habitat features in the bay are the sub-tidal rocky reefs (Fig. 2). These reefs harbour the vast majority of the Bay’s biodiversity [56] and are important to commercial and recreational fisheries [57]. The rocky reef habitat is restricted to the shallow periphery of the Bay with each discrete reef isolated by a matrix of unconsolidated mud and sand. For the purpose of this study, we used a subset of eight individual reefs (Fig. 2) to explore the relative importance of individual biological and physical drivers to population connectivity.

The hydrodynamics of PPB have been well studied and accurately modelled since the mid 1980′s [58] accompanied by a long history of in situ measurements (salinity, temperature, etc.) and ecosystem monitoring [59], resulting in an accurate and well validated representation of the Bay’s dynamics. For this study, we used a PPB-wide 3-dimensional hydrodynamic model (400 m horizontal resolution, 8 vertical layers, and hourly time-steps, [60]) incorporating wind, sea level, temperature, air pressure, tides, and solar radiation forcing [61]. The hydrodynamics, together with high-resolution rocky reef habitat data [62], represent PPB’s physical domain, within which the simulated individual dispersers interact.

An individual-based model of dispersal (modified from [63]) was used to simulate the movement and settlement of dispersers throughout PPB. Although this numerical approach has been used successfully in quantifying passive fish dispersal with simplified biology (e.g., [64, 65]), we have made several novel advancements to more accurately represent the reality of key biological traits typical of the larval life histories of marine invertebrates and fishes. First, we have implemented a larval growth scheme in which all biological parameters related to development are capable of changing through time, allowing individuals to change behaviour and sensing capacity, as well as become less susceptible to physiological stress (resulting in decreased mortality over time), for example. Second, we have added a suite of behavioural schemes to match the known and hypothesised movement patterns (e.g., diel vertical migration, homing behaviour) of marine taxa [51]. All key biological parameters (Table 1) important to population connectivity and their function in the growth and behavioural schemes are described below.

**Table 1:** Model input parameters of interest used in the sensitivity analysis for the Port Phillip Bay marine population connectivity model

Parameter	Description	Value range
RO	Reproductive output (larvae) per unit area	[100, 10,000]
A	Daily larval mortality (Weibull rate parameter)	[0.01, 0.50]
B	Daily larval mortality (Weibull shape parameter)	[0.50, 1.0]
PreP	Proportion of maxPLD required for competency	[0.05, 0.95]
ComR	Rate of transition to being competent for settlement	[0.05, 0.50]
DevP	Initial relative developmental time as passive w/initFV	[0.05, 0.95]
iFV	Fall velocity during DevT (ms^−1^, positive up)	[−0.001, 0.001]
K	Diffusivity, or the biological-physical repulsion among larvae (m^2^s^−1^)	[0.01, 1.00]
Behav	Vertical behaviour strategy: Passive, Benthic-seeking (1), or Diel migration (2).	[0, 1, 2]
Sp	Behaviour parameter: Swimming capacity Vertical swim speed is scaled at 5 % of this. (ms^−1^)	[0.001, 0.100]
TD	Behaviour parameter: Target depth (m)	[0.5, 20.0]
HmD	Behaviour parameter: Habitat detect distance (km)	[0, 2, 4, 6, 8, 10]
PLD	Maximum duration of larval stage (days)	[1, 50]
S_r_	Post-settlement survival prior to recruitment	[0, 1]
rf	Unique reefs within PPB system (ID)	[3, 10, 12, 16, 18, 23, 26, 32]
rls	Date of larval release	[July1 2009, October 1 2009, & January 1 2010]

Intrinsic parameter value ranges were chosen to be as broad as possible, but still biologically realistic, in order to capture most of potential variability in early life histories among benthic marine organisms. Reefs and larval release dates were chosen to capture the full range of geographical and temporal variability in local oceanography

At the initiation of emigration, the reproductive output (RO) is determined by the fecundity and abundance of the modelled taxon, and the habitat characteristics of the natal site. While individuals move through the environment (Transport and movement stage, Fig. 1), their trajectories and success are governed by mortality, growth and development, and individual-based behavioural parameters. Mortality was modelled as a Weibull function with a rate (A) and shape (B) parameter, capturing a full range of empirically-based mortality functions (e.g., [52]) due to processes such as natural mortality and starvation. This function has the capacity to represent the exponential decay function common to most dispersal models (if B = 1, then the Weibull reduces to the exponential function), and the flexibility to represent a ‘fat tail’ dispersal kernel, which is perhaps more appropriate for many taxa [50]. Individual growth and development is controlled through four parameters: 1) precompetency period (PreP) in which individuals are not physiologically capable of settlement; 2) competency rate (ComR) describing the developmental transition to competency; 3) development period (DevP) as a proportion of the precompetency period within which the individuals are passive dispersers before the onset of active behaviour; and 4) the initial fall velocity (iFV) describing the buoyancy of the individuals during the early development period. The flexibility in this growth scheme has the capacity to represent a broad range of taxa, including drifting seaweeds, slowly developing fish larvae and quickly developing invertebrate larvae (e.g., species with non-feeding larvae). Following the developmental period, individuals have the capacity to move, implemented through three potential behavioural strategies: 1) passive strategy where the individuals are transported by currents only; 2) benthic seeking strategy where individuals actively and constantly swim (with speed, Sp*0.05) to a specific target depth (TD); and 3) diel vertical migration where individuals actively swim (at speed, Sp*0.05) to a target depth (TD) only during daylight hours. Concurrently with these vertical swimming strategies, all non-passive individuals have the capacity to sense and swim (at speed, Sp) to nearby habitat patches at a given detection limit or homing distance (HmD) from individual reefs, to simulate the typical distances over which larvae have the potential to detect and orientate to benthic settlement habitat using auditory and/or olfactory cues [66]. Finally, following successful transport and movement through the environment, competent individuals may settle to suitable reef habitat patches and a proportion of these survive to transition to the final stage of recruitment. Post-settlement mortality rates in benthic marine organisms are highly variable, but can be as high as 96 % within the first 24 h [67]. As such, post-settlement survival was determined by a simple survival probability (S_r_), allocated randomly across patches to represent (unknown) recruitment costs [49].

Simulations were completed for each of eight selected reef habitat patches to explore the impact of geographic location on connectivity outcomes. In addition, simulations were initiated on three separate dates (rls, Table 1) to capture temporal variability across three seasons and representing potential spawning dates for different taxa, while maintaining computational feasibility. In this way, we were able to quantify the relative impact of both intrinsic (e.g., growth, behaviour) and extrinsic (currents, habitat quality) factors, and their geographic and temporal signatures, on 10 different ecologically-relevant metrics of population connectivity that span three scales and a range of questions. At the local scale we calculated the proportion of individuals released at initiation that recruit back to the natal habitat patch, termed local retention (LR), as well as the proportion of successfully recruiting individuals that originated from the focal patch, or self-recruitment (SR). In addition, we calculated the diversity of successful settlers (H’) to each habitat patch, which may influence population persistence and long-term resilience [24]. At the scale of downstream connectivity, we quantified several distance-based measures of population connectivity, including the median geographic distance displaced by individuals (mdG) and the maximum distance (mxG), as well as the total proportion of successful settlers to all downstream patches (S). The downstream contributions from each source (i.e., source strength) was quantified by counting the number of downstream linkages (dC) and calculating the weighted degree centrality (C_w_), a network-based measure characterising the strength and evenness of downstream linkages [68]. Finally, at the eight-patch metapopulation scale, we calculated the metapopulation growth rate [λM, 6] and the metapopulation capacity [λmax, 5] (see Table 2 for additional details on the ecological significance of each metric).

**Table 2:** Model output variables and descriptions used in the sensitivity analysis

Variable	Description
Per reef patch	
I. Local settlement	
LR^a^	Local retention
SR	Self-recruitment (with eight-patch metapopulation)
H’	Shannon index of diversity of settlers (sensitive to weak connections)
II. Downstream connectivity	
mdG^a^	Median geographic distance of downstream connections
mxG	Maximum distance of downstream connections
S	Total proportion of larvae that settle downstream
dC	Out-degree, total number of downstream connections
C_w_^a^	Weighted degree centrality as dC^(1-α)^ x S^α^; dp is d as proportion of total possible connections; α = 0.5
III. Metapopulation consequences	
λ_M_^a^	Metapopulation growth rate with variable population sizes, fecundity, & survival [6]
λ_max_	Metapopulation capacity [5]

Selected parameters (marked with ^a^) are presented in the Figures, with the remaining in the Additional file 1. The intrinsic and extrinsic drivers of larval dispersal Fig. 1, (Table 1) can influence population connectivity at three different scales. At a local scale, the magnitude of local settlement will depend on: (1) what proportion of locally spawned larvae are retained and settle to their natal reef (local retention); (2) what proportion of settling larva were spawned locally (self-recruitment); and (3) whether dispersing larvae come from a diversity of sources (Shannon H’). At a regional scale, how strongly connected populations are by larval dispersal will depend on: the distribution of dispersal distances (mdG and mxG), what proportion of spawned larvae survive to settle to another reef (S), how many downstream reefs receive these larvae (dC) and whether the strengths of these connections are even or skewed (C_w_). At a metapopulation scale, connectivity patterns have important consequences for rates of replenishment (i.e., growth) across all patches (λ_M_) and the ability of a species to persist in the landscape/seascape (λ_max_)

### Importance analysis

To quantify the main effects and all interactions, a global sensitivity analysis (GSA) was used [69, 70]. Due to the computational requirements and complexity of the PPB connectivity model, we performed a non-parametric regression tree GSA on a meta-model [69, 71] derived from a full suite of input parameters to quantify parameter importance (R package CompModSA with ‘sensitivity’ function). To aid in the interpretation of these importance values, and to effectively quantify the main effect and direction of influence of parameters on model output, we paired this with a generalized linear regression (GLM) analysis [72]. For the GLM, we calculated the main effects on the standardized data using the identity link function. Sensitivities where visualized by plotting the effect of one standard deviation change in each model parameter on the response [72]. For computational feasibility, we ran the connectivity model for a suite of parameter values generated using an optimum Latin Hypercube Sample (LHS) scheme [73], assuming each parameter has a uniform distribution. The LHS scheme ensures that the entire multivariate range is sampled and the full behaviour of the model explored. The PPB connectivity model was used to simulate 21,600 scenarios, representing 900 unique parameter combinations (each containing values for all 13 parameters) for each of eight habitat patches across three release dates (seasons). The complete ensemble of simulations consisted of 2,583 million dispersers being tracked (~130,000 individuals per simulation) for the full sensitivity analysis. The parameter importance was based on the total sensitivities on the model output variables (Table 2) and a bootstrap technique was applied to characterise uncertainty.

## Results

**Fig. 3:**
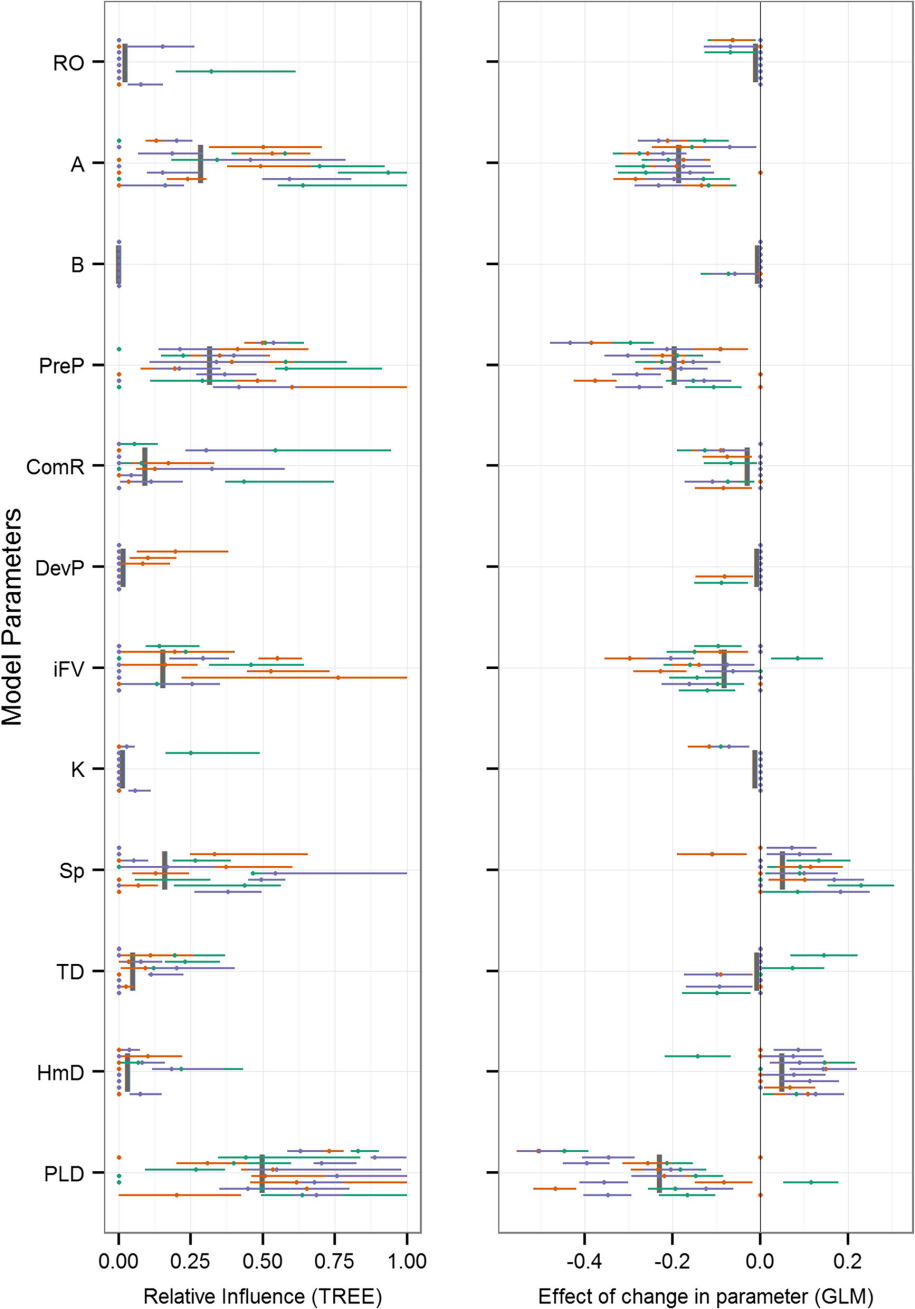
Model sensitivity for local retention (LR). Two-panel plot of the influence of model parameters (y-axis) on local retention. The regression tree GSA relative influence (left) and generalized linear regression beta coefficients (right) are plotted for all reefs (individual horizontal bars spread vertically in each parameter’s row) and release times (unique colours within each reef’s horizontal bar). Parameter means are shown as grey vertical bars

**Fig. 4:**
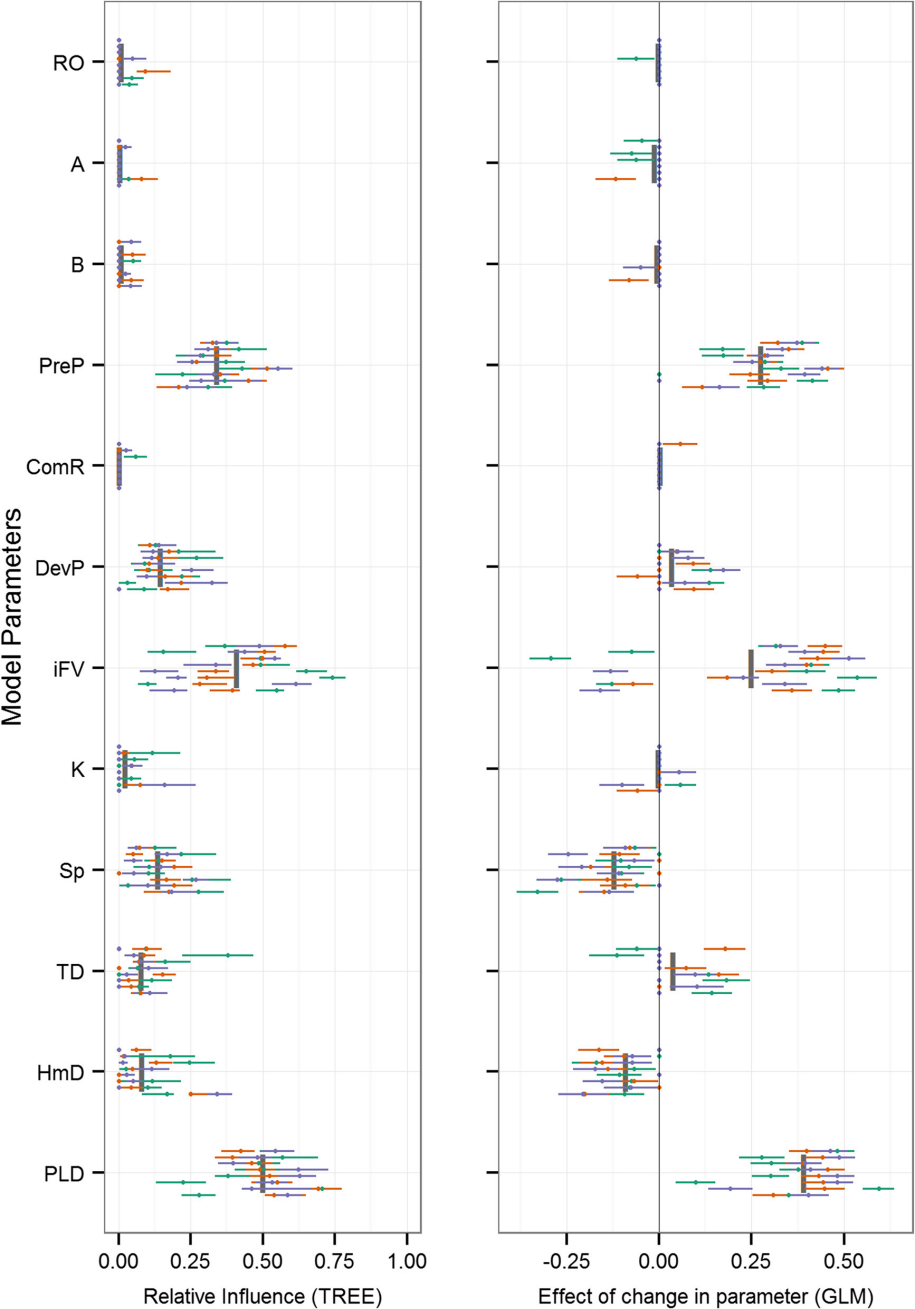
Model sensitivity for median geographic distance (mdG). Two-panel plot of the influence of model parameters (y-axis) on median geographic distance. The regression tree GSA relative influence (left) and generalized linear regression beta coefficients (right) are plotted for all reefs (individual horizontal bars spread vertically in each parameter’s row) and release times (unique colours within each reef’s horizontal bar). Parameter means are shown as grey vertical bars

**Fig. 5:**
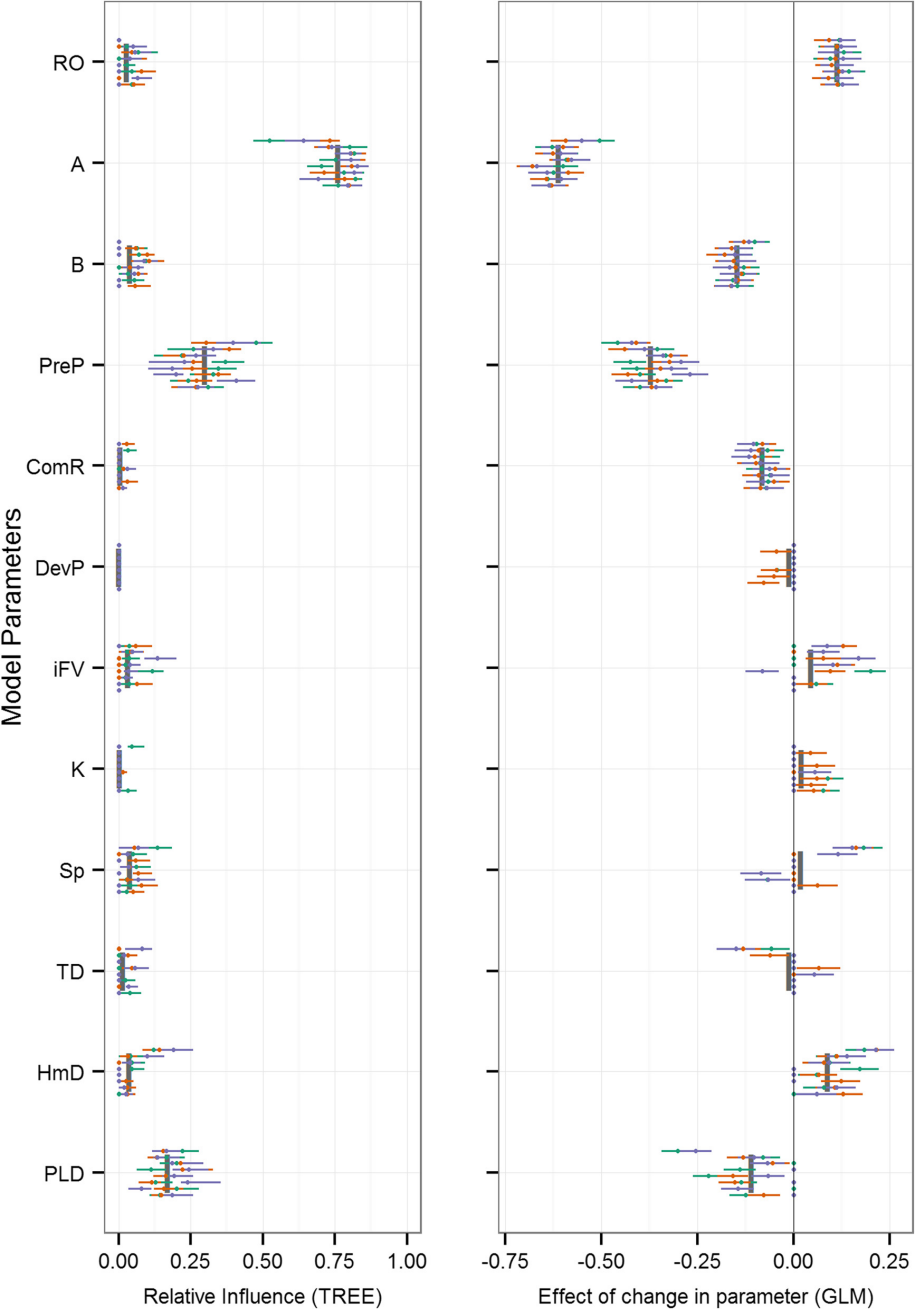
Model sensitivity for downstream degree centrality (C_w_). Two-panel plot of the influence of model parameters (y-axis) on downstream degree centrality. The regression tree GSA relative influence (left) and generalized linear regression beta coefficients (right) are plotted for all reefs (individual horizontal bars spread vertically in each parameter’s row) and release times (unique colours within each reef’s horizontal bar). Parameter means are shown as grey vertical bars

**Fig. 6:**
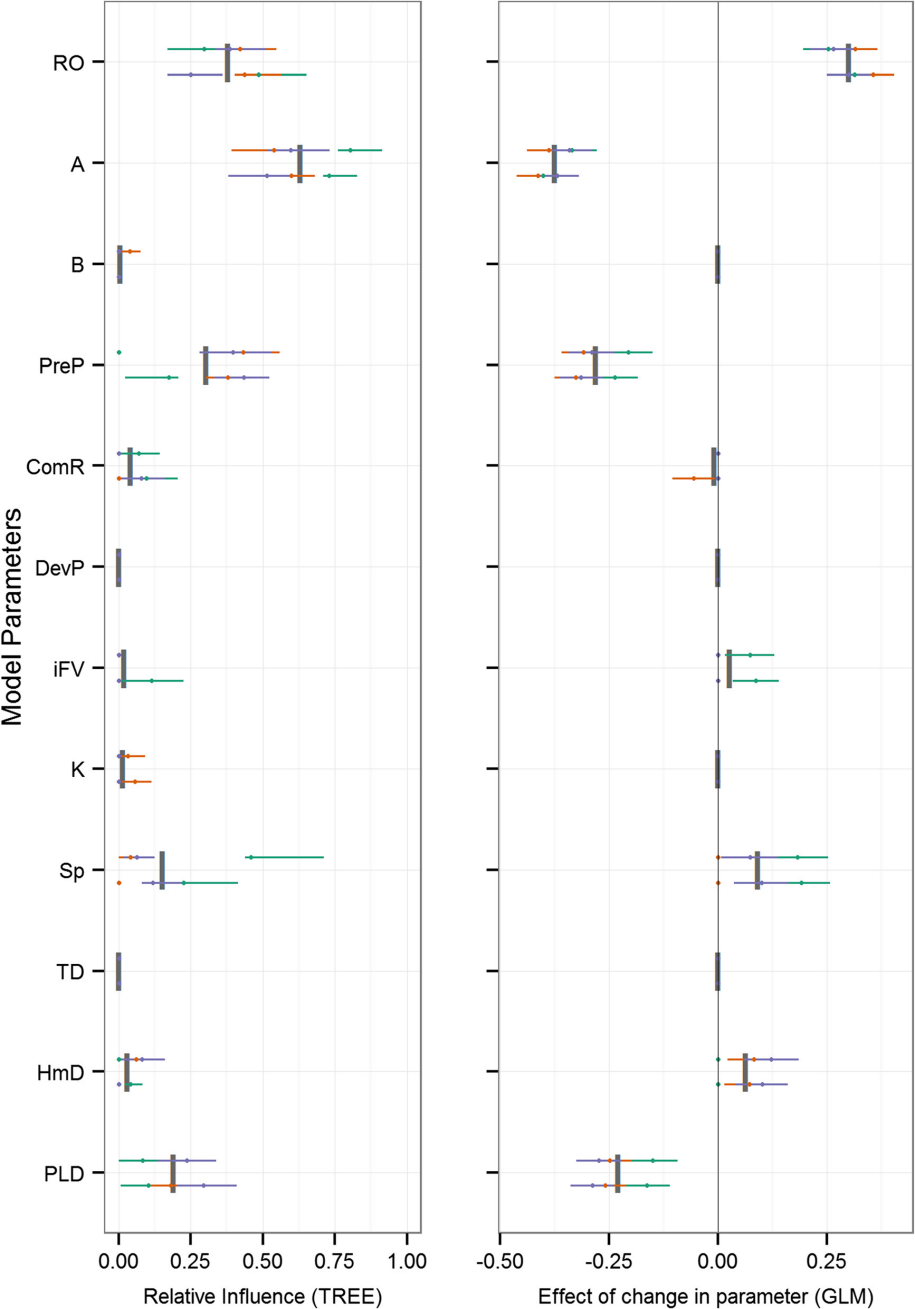
Model sensitivity for metapopulation growth rate (λ_M_). Two-panel plot of the influence of model parameters (y-axis) on metapopulation growth rate. The regression tree GSA relative influence (left) and generalized linear regression beta coefficients (right) are plotted for all reefs (individual horizontal bars spread vertically in each parameter’s row) and release times (unique colours within each reef’s horizontal bar). Parameter means are shown as grey vertical bars

The mean relative influence and 95 % confidence limits were plotted for parameters using the LHS (each with 900 parameter sets) for all eight reefs and three release dates (Figs. 3, 4, 5 and 6). The mean variance explained (R^2^) in the recursive partitioning regression analysis for each response variable and the global mean relative influence of each parameter is presented in Table 3, and plotted as vertical grey bars in Figs. 3, 4, 5 and 6. Of the 10 connectivity response variables, four are presented: local retention (Fig. 3), median geographic distance (Fig. 4), weighted downstream degree centrality (Fig. 5), and metapopulation growth rate (Fig. 6), with the remaining six in the Additional file 1. The importance analysis was completed across all simulations for each vertical behaviour strategy (Behav, Table 1) independently to quantify any behavioural-specific responses. The relative parameter importance across all behaviour strategies were strongly consistent and therefore all results presented do not separate benthic-seeking from diel vertical migration. The relative importance of larval behaviour is then quantified in the swimming speed (Sp), target depth (TD), and homing distance (HmD) parameters.

**Table 3:** Mean importance values for each model input parameter across all ensembles (values plotted as grey vertical bars in Fig. 3)

Parameter	LR^*^	SR	H	mdG^*^	mxG	S	dC	Cw^*^	λ_M_^*^	λ_max_	Mean
R^2^	0.66	0.80	0.84	0.86	0.89	0.88	0.88	0.89	0.79	0.81	0.83
RO	0.02	0.01	0.01	0.01	0.00	0.01	0.18	0.03	0.38	0.00	0.07
A	**0.28**	0.05	0.20	0.01	0.05	**0.70**	**0.56**	**0.76**	**0.63**	**0.46**	0.37
B	0.00	0.03	0.09	0.01	0.03	0.01	**0.13**	0.04	0.01	0.02	0.04
PreP	**0.32**	**0.39**	**0.16**	**0.34**	0.07	**0.30**	**0.20**	**0.30**	**0.30**	**0.42**	0.28
ComR	0.09	0.02	0.01	0.00	0.00	0.01	0.00	0.01	0.04	0.07	0.03
DevP	0.02	0.06	0.07	0.15	0.06	0.00	0.02	0.00	0.00	0.00	0.04
iFV	**0.15**	**0.29**	**0.15**	**0.41**	**0.18**	0.02	0.08	0.03	0.02	0.04	0.14
K	0.01	0.03	0.02	0.02	0.01	0.01	0.01	0.00	0.02	0.06	0.02
Sp	**0.16**	**0.17**	**0.42**	**0.14**	**0.26**	**0.15**	**0.28**	0.04	**0.15**	0.09	0.19
TD	0.05	0.10	0.07	0.08	0.05	0.03	0.02	0.01	0.00	0.02	0.04
HmD	0.03	0.08	0.07	0.08	0.07	**0.15**	0.02	0.04	0.03	0.04	0.06
PLD	**0.50**	**0.56**	**0.55**	**0.50**	**0.67**	**0.18**	**0.32**	**0.17**	**0.19**	**0.50**	0.41

Importance values greater than 10 % are bolded. The mean R^2^ values from the regression tree analysis are reported per response variable in the first row. Selected parameters (marked with *) are presented in the Figures

Across all response variables, high variability resulted from both the geographic context and timing of initiation. Variation driven by the geographic location of the eight reefs is evident as the horizontal displacement when comparing individual bars (reefs) across the vertical extent of each parameter’s row. Similarly, the variation resulting from different release times is seen as the total spread across colours (release dates) along each horizontal plane (unique reefs). Importance values for all simulations are shown, and those that are not significant are plotted as points along the y-axis and some points and lines may fall on top of each other. Generally, across the majority of response variables, larval mortality rate (A) and the length of the pelagic larval stage (PLD) were the most influential parameters (Figs. 3, 4, 5 and 6, Table 3). For the metapopulation growth rate (Fig. 6), reproductive output (RO) outweighed PLD in importance. At the next level of importance, the extent of the precompetency period (PreP) was consistently identified as being an important parameter across all response variables. A suite of parameters displayed highly variable levels of importance across most response variables, including the initial fall velocity (iFV), and swimming speed (Sp). Three parameters were consistently of low importance (HmD, DevP, and TD), with the remaining parameters having minimal influence on population connectivity (ComR, K, B).

Focusing on the local patch scale, retention of individuals within the natal site (Fig. 3, Table 3) increased primarily with a decrease in PLD, a decrease in the precompetency period (PreP), and a decrease in larval mortality (A), with levels below 10 % across all reefs (Additional file 2: Table S1). At local scales, the relative influences of these parameters were somewhat more idiosyncratic when settler diversity (H) and levels of self-recruitment (SR) were considered. Distance-based measures of downstream connectivity consistently ranked PLD as the most influential parameter (distance increased with increasing PLD), followed by the initial fall velocity (iFV), swim speed (Sp), and precompetency period (PreP). The distribution of downstream connectivity (number of connections, dC, and the degree centrality, C_W_) was determined primarily by larval mortality (A), followed by the precompetency period (PreP), swim speed (Sp), PLD, and to a lesser extent, reproductive output (RO). The proportion of successful settlers was driven by mortality rate (A), followed by the precompetency period (PreP), with swimming speed (Sp), PLD, and homing distance (HmD) all with modest influences. At the metapopulation scale, larval mortality rate (A), the precompetency period (PreP), and the reproductive output (RO) were the most influential drivers, followed by the PLD and swim speed (Sp).

Across the majority of population connectivity response variables the following six parameters consistently had low relative influences: larval mortality shape parameter (B), rate of transition to competency (ComR), initial development time (DevP), bio-physical cohesion (K), the target depth (TD), and homing distance (HmD). The strong influence of geographic location or seascape context was clear when comparing values in the response variables across all reefs (Additional file 2: Table S1).

## Discussion

The unique characteristics of connectivity in marine and aquatic environments, such as the early developmental stages of dispersers and the strongly advective environment, require a more biologically-physically balanced and comprehensive framework to study the importance of individual parameters to this complex process. Here, we have suggested an alternative conceptualisation of connectivity which integrates recent advances across systems and taxa (Fig. 1) and provided insights from applying this framework to the analysis of potential connectivity in Port Phillip Bay, Australia. Although the outcomes presented are from a large semi-enclosed bay, the relative importance of bio-physical parameters would be expected to scale up to broader scales and be relevant for other marine systems. Indeed, the importance of PLD, mortality and behaviour are consistent with many other study systems [40, 38, 74, 36, 75, 19, 76], and the importance of geographic and temporal context is only beginning to be recognised [77, 78].

In particular, the strong temporal and geographic variability evident across all parameters and connectivity metrics (Figs. 3, 4, 5 and 6) may be both a unique consequence of dispersal in the ocean, and relatively unrecognized in the study of population connectivity [79]. The geographic location of individual reefs (Fig. 2) and the surrounding hydrodynamic environment had a large impact on connectivity outcomes (Additional file 2: Table S1), yet strong coherence remained in the relative importance of bio-physical parameters. For example, reef #16 is most isolated (low SR, Additional file 2: Table S1), whereas the nearest neighbours, reefs #18 and #12, are strong sources of dispersing individuals to many patches throughout the Bay (high S, mdG, and mxG). Despite the semi-enclosed nature of the bay, the system is generally well connected for many modelled parameter combinations (mean mxG across all reefs is 0.81, 81 % of total possible downstream connections are made on average). Similarly, the timing of spawning (i.e., initiation) had considerable impact on connectivity outcomes, evident in the wide horizontal spread in importance values across many parameters and connectivity outcomes (Figs. 3, 4 and 5). This strong spatial and temporal variability resulting from the geographic and temporal context suggests predicting demographic consequences or local-scale patterns in marine environments from generalised ‘dispersal syndromes’ [20, 21] may be challenging for benthic species with pelagic larvae where the environment can exert considerable influence on dispersal patterns [79]. Our results suggest that even when species exhibit stereotypical behaviours during early development, local oceanographic conditions can interact with such behaviours to result in fundamentally different dispersal outcomes among locations and release times.

Despite this spatiotemporal variability, broad patterns in the dominant drivers of connectivity outcomes were apparent and strongly consistent. Across the majority of response variables (Table 2), three parameters were consistently the most influential on population connectivity across scales: 1) the mortality rate during the dispersal phase, 2) the maximum duration of the pelagic larval stage (PLD), and 3) the relative duration of the pre-competency and competency windows of dispersers. The importance and main effect of mortality rate (Figs. 3, 4, 5 and 6) on connectivity is intuitive and matches empirical data (e.g., [52]), theoretical studies (e.g., [36]), and expectations based on dispersal syndromes (e.g., [22]). Although the importance of PLD in marine systems has been well recognized [50, 38], the main effect in several connectivity outcomes is less so. Here, an increase in PLD increased the connectivity distance (mdG, mxG) and number of connections (dC), yet decreased total settlement (S), local retention (LR) and self-recruitment (SR), and resulted in negative impacts on metapopulaton growth rate and capacity (λ_M_, λ_max_, respectively). Increasing the time spent dispersing effectively increases the likelihood of making some long-distance connections, while at the same time, increases the likelihood of being lost at sea resulting in larval wastage. Quantifying these mixed effects of PLD on connectivity outcomes could help disentangle the equally mixed results present in the empirically-based, albeit indirect, correlations between PLD and genetic distance or gene flow [80].

The relative length of the precompetency period (PreP), a trait unique to marine and aquatic taxa, was the third most important parameter. Increasing the precompetency period decreases the potential settlement window, effectively increasing the geographic distance (mdG and mxG) individuals travel while decreasing the proportion that settled locally (LR, SR). These parameters have been highlighted in the past as being relatively important [32, 40], yet the scale-dependence and effect highlighted here are new. Delaying settlement competency until later in the transport stage causes more individuals to be swept away from their natal habitat patch (lower LR), many not finding suitable habitat (decreases dC, C_W_) and effectively decreasing metapopulation growth (λ_M_, λ_max_). Although relatively little is known of the development transition phase of many marine species (but for corals see [52, 81]), it is growth dependent and sensitive to temperature [82], therefore having obvious implications under future warming scenarios [83, 10].

A suite of parameters were identified as having an intermediate level of influence, often with inconsistencies in the strength and direction of effect on connectivity (right panels in Figs. 3, 4, 5 and 6). These parameters include the initial fall velocity (iFV), swimming capacity (Sp), distance at which individuals can sense suitable reef habitat (HmD), and the reproductive output (RO). The remaining parameters explored had consistently low influence on the connectivity outcomes (B, ComR, K, and TD). Across these low and intermittently important parameters, there was a high level of variability in the direction of impact, making it difficult to identify direct causal relationship.

Considering the proposed four stages of marine population connectivity (Fig. 1), our sensitivity analysis suggests that the settlement stage (PLD, competency, and habitat structure) and the transport and movement stage (mortality and currents) are the most critical drivers of connectivity outcomes in Port Phillip Bay. The initiation of the emigration stage is important in metapopulation-wide measures, which are largely driven by reproductive output (fecundity and abundance of habitat patches). Increased reproductive output also leads to a greater magnitude in connection strengths throughout, although this is not reflected in the relative measures presented here. Recruitment, the final stage of population connectivity, had relatively little influence on connectivity outcomes, compared to the other stages. This was somewhat surprising as post-settlement mortality was allowed to vary between 0 and 100 % on a patch-by-patch basis per simulation. Implicit in our approach to post-settlement mortality is the assumption that the specific cause of increased mortality is acting at, or below, the patch-scale (e.g., poor habitat quality, increased predators) and is not spatially autocorrelated, such as some disturbance events (e.g., storms and urchin outbreaks). Although these added complexities were beyond the scope of the present study, further research is needed to elucidate how environmental heterogeneity and anthropogenic disturbances may influence recruitment and connectivity outcomes.

## Implications for future research and management

Marine larval dispersal is biophysically complex and our understanding of this process and its consequences to local and regional population dynamics and management is still limited, despite considerable research. Our findings identify several areas where future research should be targeted.

### Better estimates of the key intrinsic drivers of dispersal

Larval mortality was the most important intrinsic parameter influencing dispersal outcomes at all scales. Unfortunately, empirical measures of larval mortality under natural conditions are scarce and most marine dispersal models use guestimates based on the few published studies available [84] and often assume these rates are invariant [43]. Ecologists and larval biologists have made great strides in recent decades on amassing knowledge of larval durations and the timing of competency to settle for a diversity of marine taxa (e.g., [38]). Efforts need to be redirected to increase our understanding of mortality, particularly in how rates change as a function of age, size, or condition.

### Ground-truthing biophysical models of marine dispersal and population connectivity

Consistent with other research, dispersal outcome are highly variable across space and time. Such spatio-temporal variability makes it difficult to gain a full understanding of the dynamics of marine populations from empirical studies, which are often logistically and financially constrained to snapshots of the dispersal process in space and time. This, in combination with greater computational capability and finer-resolved hydrodynamic models, has led to an increasing focus and reliance on predictions from biophysical models. Although validation of hydrodynamic models is virtually a requirement, corroborating dispersal predictions from such models is considerably more challenging. To our knowledge, no larval dispersal model has been ground-truthed with empirical estimates of dispersal (although this has been achieved in comparisons of generalized ocean circulation with estimates of gene flow among populations (e.g., [85])). This greatly limits the confidence in modelled estimates of dispersal and connectivity. Our modelling framework provides a mechanism for model parameter tuning and validation in comparison to empirical estimates. Once ground-truthed, simulations can be run across the full spectrum of spatio-temporal variability to generate more realistic estimates of connectivity outcomes and their impacts on metapopulation dynamics.

### Applying connectivity models for marine management

Our framework has direct applicability to many marine and aquatic systems, and may assist in gaining a more holistic and integrated view of dispersal-based connectivity to aid in management. Connectivity is a multifaceted process and using a holistic framework to assess the primary drivers of dispersal and population connectivity will lead to greater insight into where best to target management efforts. We suggest that the metapopulation-based metrics, such as λ_M_ and λ_max_, should be used to help identify locations that are important contributors to overall growth rates or capacity to recover from disturbance. Identifying such keystone populations, which are likely to be well-connected populations that are important sources, has been a successful approach for informing conservation efforts in terrestrial ecosystems [86]. We suggest that, with realistic marine population connectivity data, identifying keystone populations will be equally informative in managing natural resources in marine ecosystems.

## Conclusions

Here, we have presented clear evidence on the relative importance of the transport and settlement stages to marine population connectivity, the key intrinsic drivers of larval mortality, the length of the pelagic larval phase, and the settlement competency characteristics, and the influence of the extrinsic factors of habitat geography and currents. Gaining a better understanding of these drivers and how they vary across species will greatly enhance our ability to predict contemporary connectivity patterns, will aid in our study of the evolution of larval dispersal strategies (e.g., [21]), and may guide a proactive approach to understanding species’ potential for adaptation to habitat and climate change to better inform marine environmental management.

## Additional file

Additional file 1: Figure S1. Sensitivity results for select output parameters are shown as two-panel plots of the influence of model parameters (y-axis) on remaining model output. Additional file 2: Table S1. Median values for all reef-based response variables are reported per reef patch and summarized across all reefs in system.
