# Supplementary material for: Identifying the key biophysical drivers, connectivity outcomes, and metapopulation consequences of larval dispersal in the sea
Source: Mov Ecol. 2015 Jul 15;3(1):17. doi: 10.1186/s40462-015-0045-6 (PMC4502943; doi:10.1186/s40462-015-0045-6)

Additional File 1

Supplemental materials for

Identifying the key biophysical drivers, connectivity outcomes, and metapopulation consequences of larval dispersal in the sea

Eric A Treml[1*], John R Ford[1], Kerry P Black[1], Stephen E Swearer[1]

1. School of BioSciences, University of Melbourne, Parkville, Victoria 3010, Australia.

Figure S1. Sensitivity results for select output parameters. Two-panel plots of the influence of model parameters (y-axis) on remaining model output. The regression tree GSA relative influence (left) and generalized linear regression beta coefficients (right) are plotted for all reefs (individual horizontal bars spread vertically in each parameter's row) and release times (unique colours within each reef's horizontal bar). Parameter means are shown as grey vertical bars.

Self Recruitment (SR)

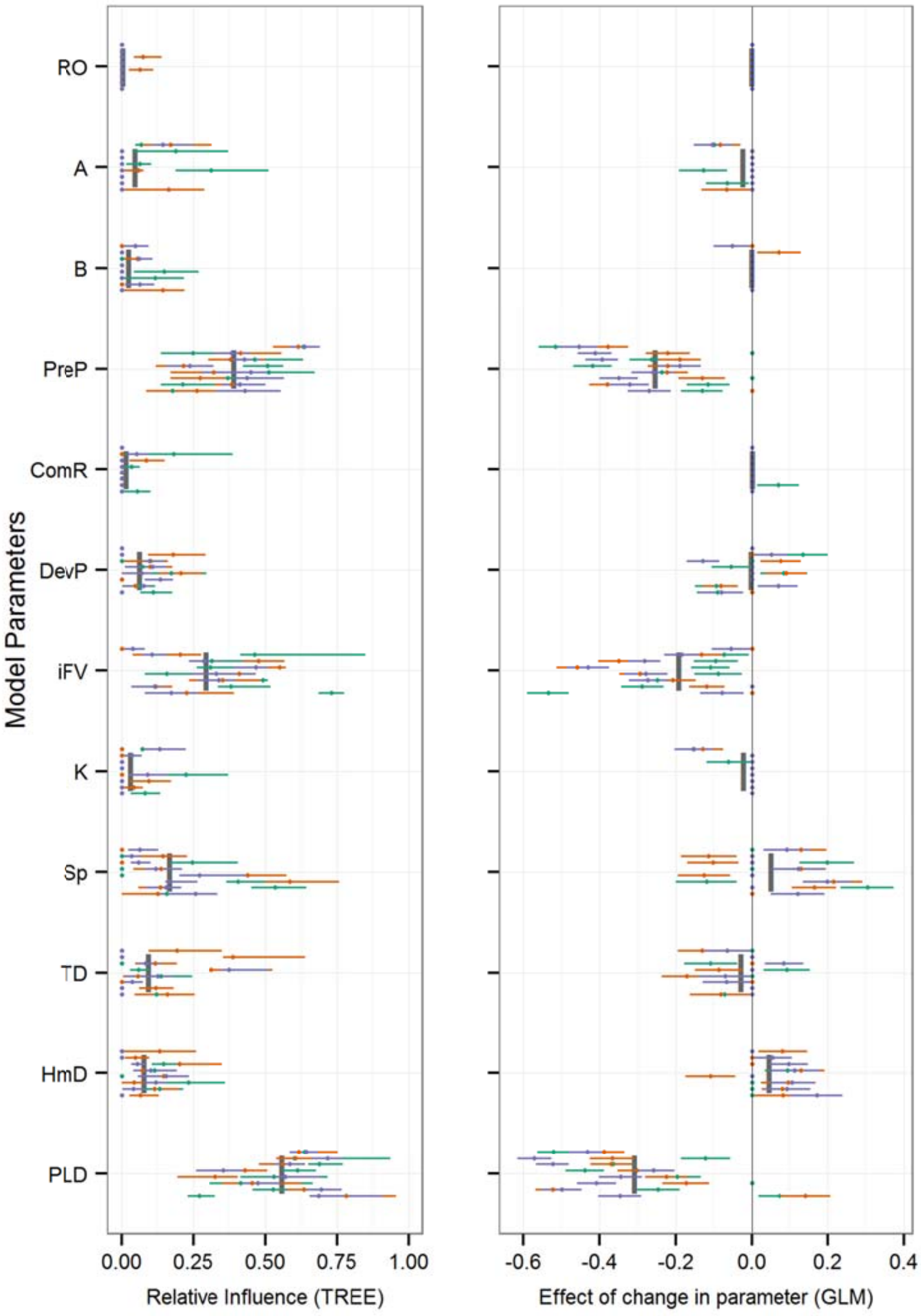

Settler Diversity (H')

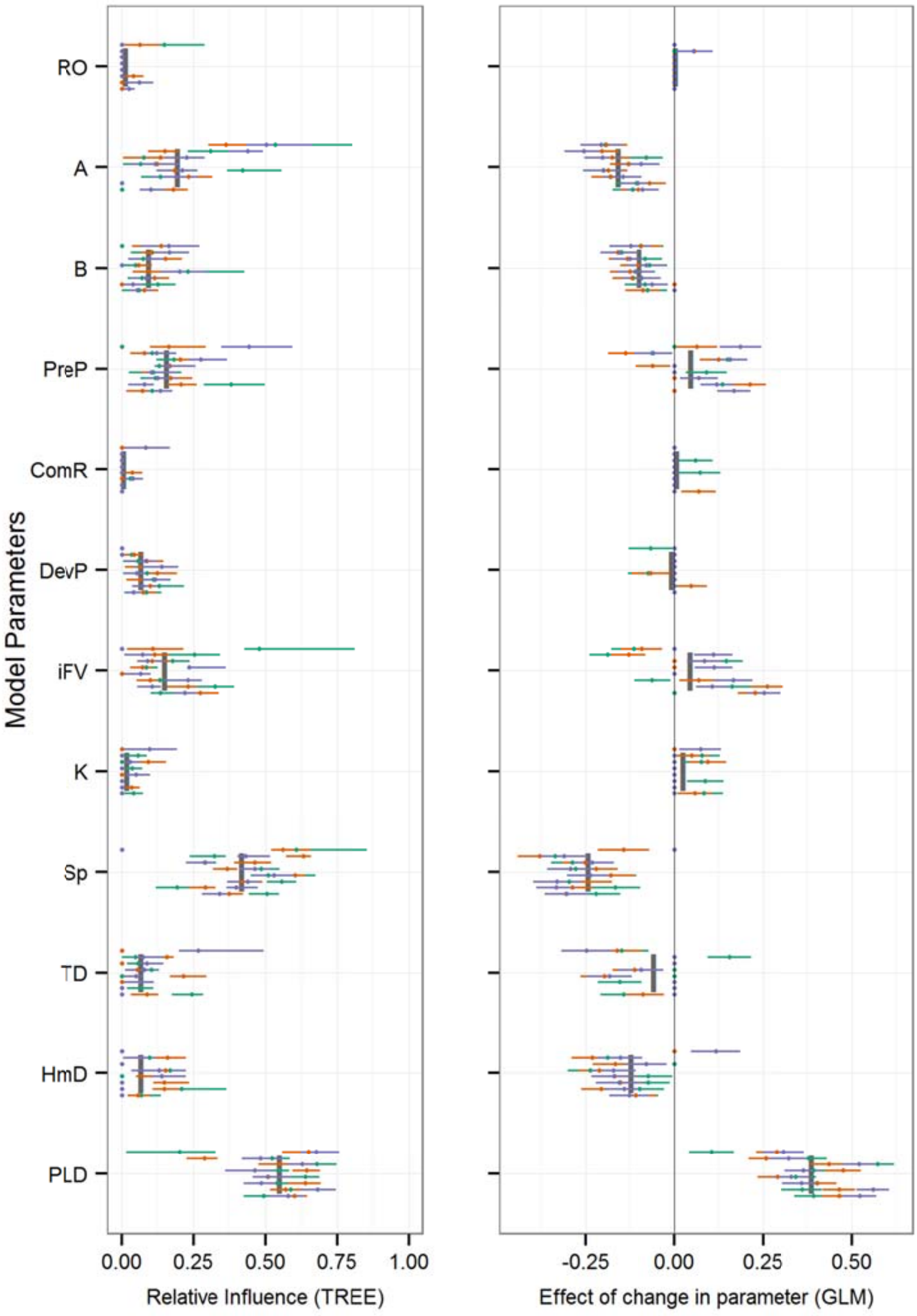

Maximum Geographic Distance (mxG)

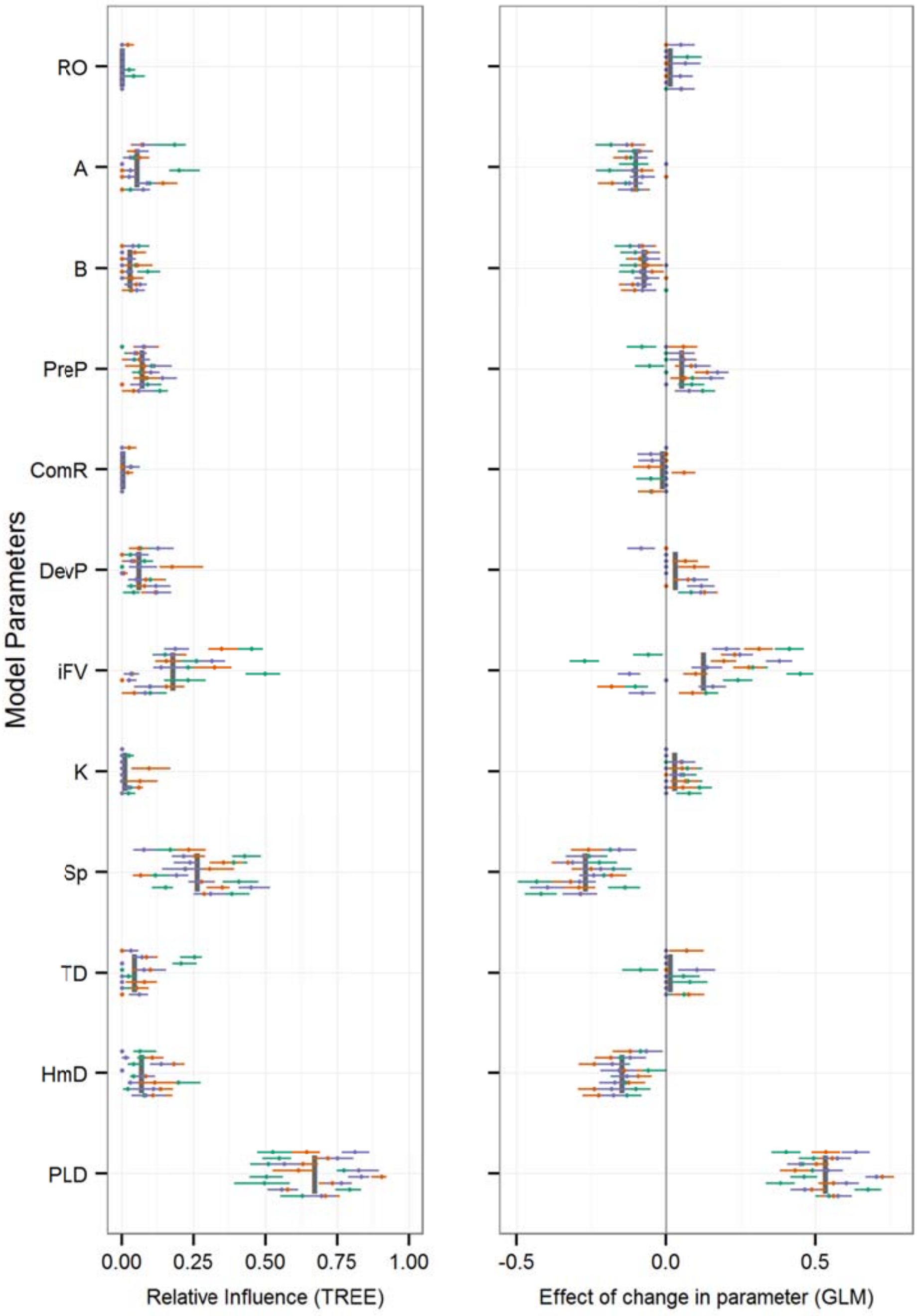

Successful Settlers (S)

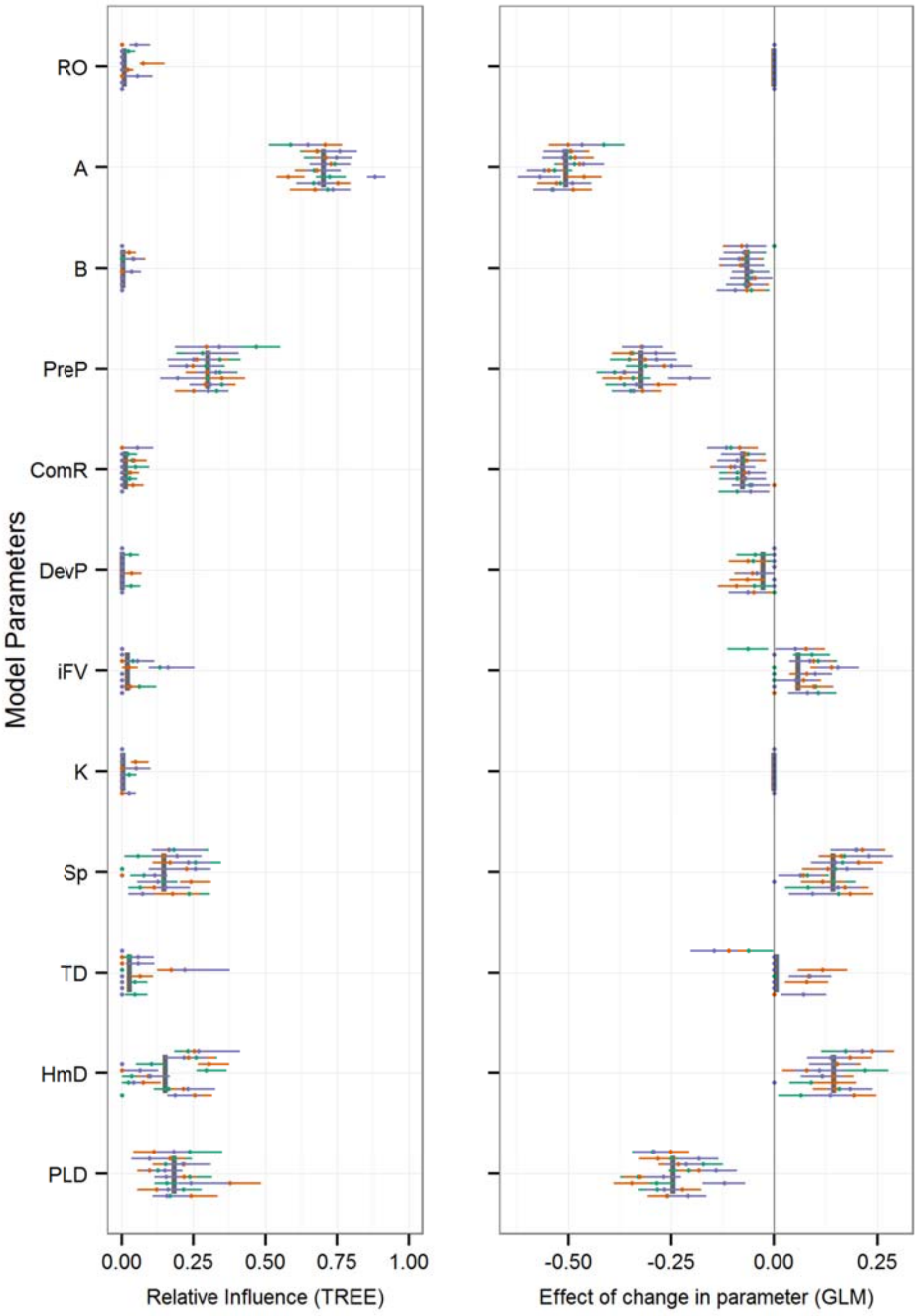

Downstream Connections (dC)

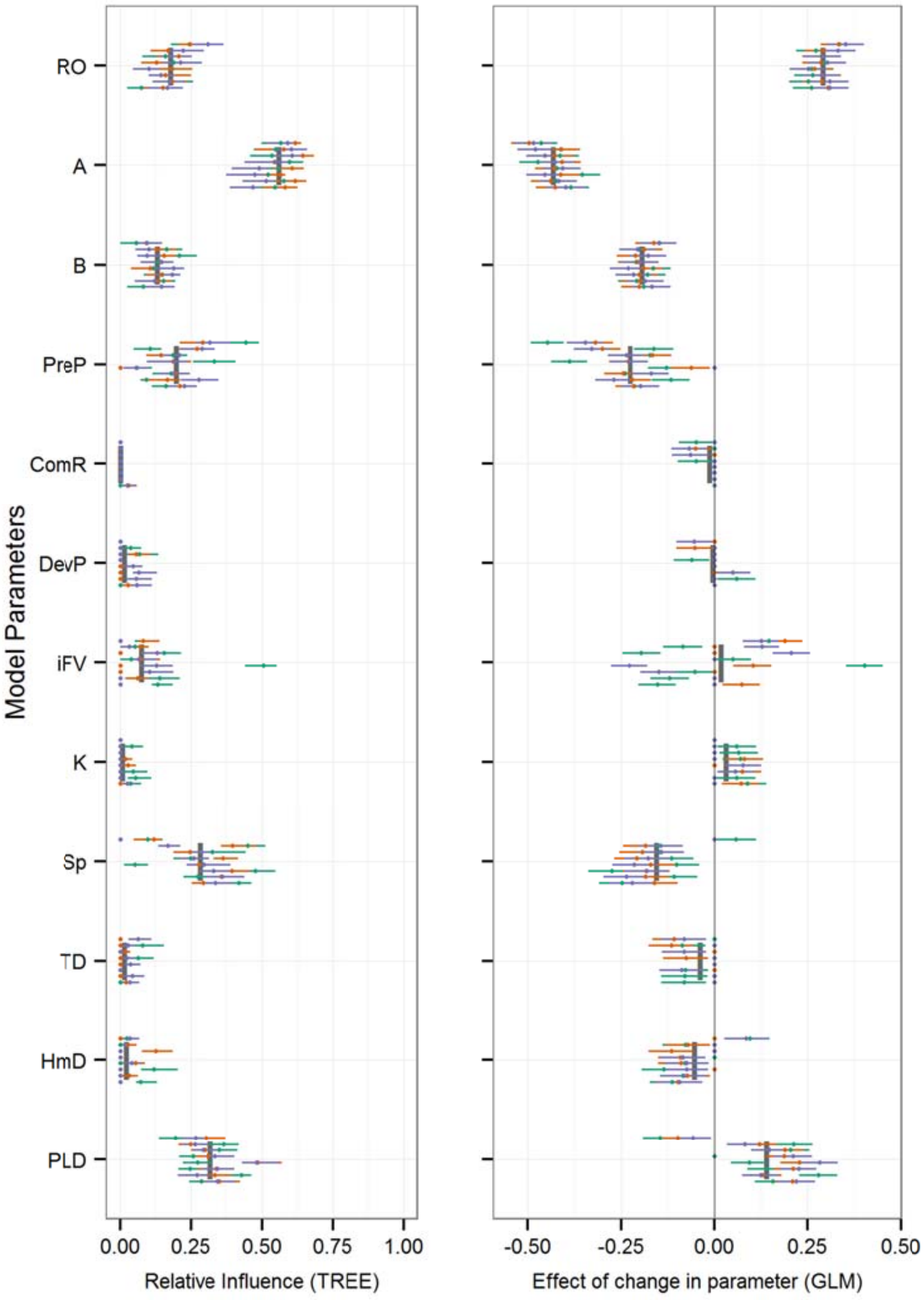

Metapopulation Capacity (λ_{max})

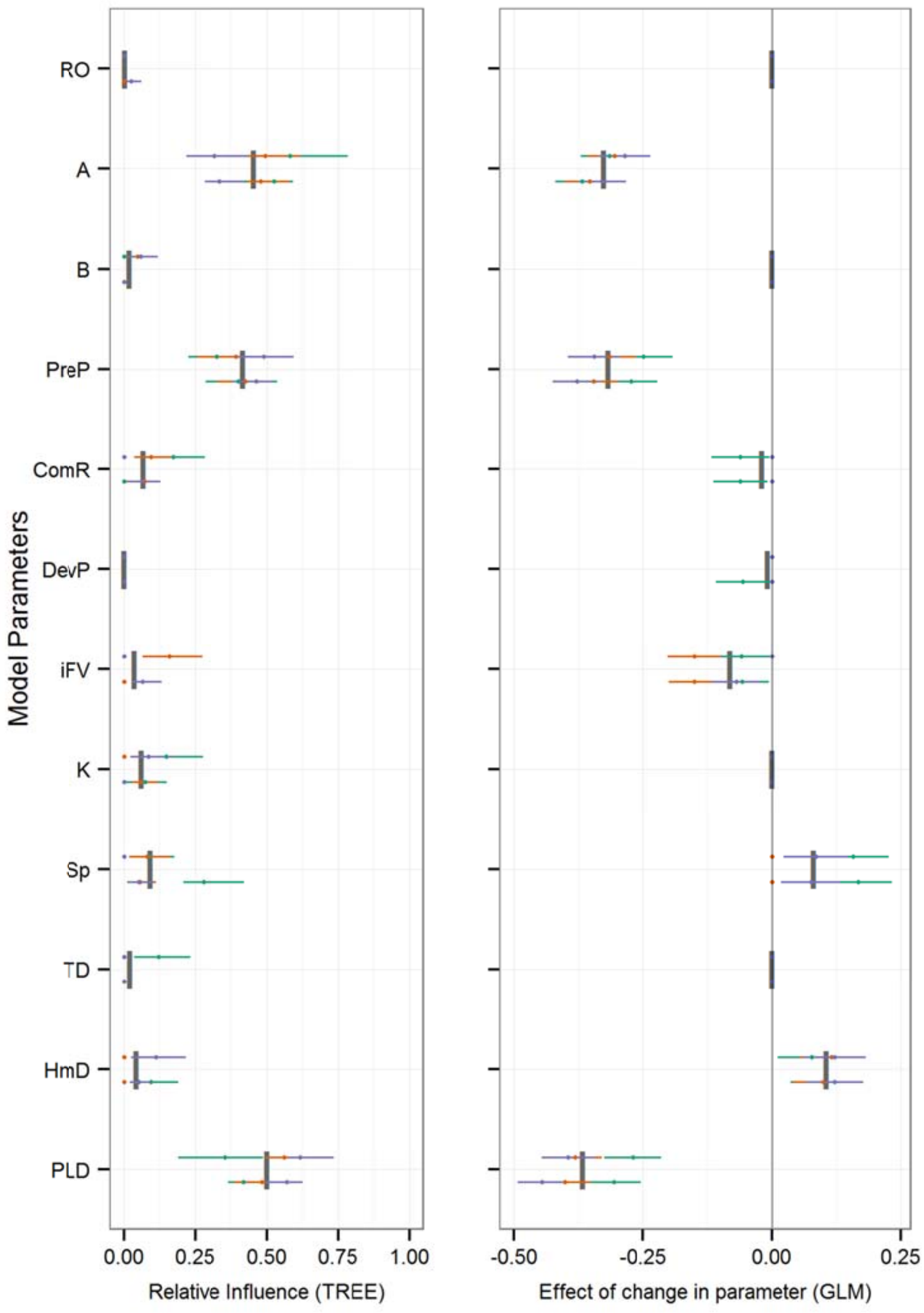

Supplement: Additional file 1: Figure S1. — Sensitivity results for select output parameters are shown as two-panel plots of the influence of model parameters (y-axis) on remaining model output. [file 40462_2015_45_MOESM1_ESM.pdf]
