# Supplementary material for: Identifying the key biophysical drivers, connectivity outcomes, and metapopulation consequences of larval dispersal in the sea
Source: Mov Ecol. 2015 Jul 15;3(1):17. doi: 10.1186/s40462-015-0045-6 (PMC4502943; doi:10.1186/s40462-015-0045-6)
Supplement: Additional file 2: Table S1. — Median values for all reef-based response variables are reported per reef patch and summarized across all reefs in system. [file 40462_2015_45_MOESM2_ESM.pdf]

Additional File 2

Supplemental materials for

Identifying the key biophysical drivers, connectivity outcomes, and metapopulation consequences of larval dispersal in the sea

Eric A Treml[1*], John R Ford[1], Kerry P Black[1], Stephen E Swearer[1]

1. School of BioSciences, University of Melbourne, Parkville, Victoria 3010, Australia.

Table S1. Median values for all reef-based response variables are reported per reef patch and summarized across all reefs in system (All), aggregated across all behavioural parameters. Lower and upper quartile values are also shown (Q1 - Q3).

**Parameter**	**Reef 3**	**Reef 10**	**Reef 12**	**Reef 16**	**Reef 18**	**Reef 23**	**Reef 26**	**Reef 32**	**All**
**LR**	0.001 (0.000-0.070)	0.003 (0.000-0.142)	0.000 (0.000-0.043)	0.001 (0.000-0.062)	0.000 (0.000-0.097)	0.004 (0.000-0.204)	0.000 (0.000-0.015)	0.001 (0.000-0.170)	0.000 (0.000-0.110)
**SR**	0.303 (0.000-0.988)	0.238 (0.000-1.000)	0.005 (0.000-1.000)	0.988 (0.000-1.000)	0.166 (0.000-1.000)	0.480 (0.000-1.000)	0.041 (0.000-1.000)	1.000 (0.000-1.000)	0.287 (0.000-1.000)
**H**	0.896 (0.000-1.736)	0.736 (0.000-1.630)	0.546 (0.000-1.738)	0.000 (0.000-1.267)	0.751 (0.000-1.753)	0.765 (0.000-1.676)	0.580 (0.000-1.614)	0.000 (0.000-0.663)	0.499 (0.000-1.676)
**mdG**	0.409 (0.014-1.189)	0.400 (0.037-0.795)	0.800 (0.206-1.266)	0.059 (0.043-1.026)	0.839 (0.016-1.273)	0.212 (0.013-1.228)	0.651 (0.228-1.571)	0.211 (0.010-1.166)	0.459 (0.016-1.266)
**mxG**	0.780 (0.086-1.000)	0.488 (0.124-1.000)	0.940 (0.392-1.000)	0.821 (0.047-1.000)	0.937 (0.011-1.000)	0.684 (0.109-1.000)	0.845 (0.330-1.000)	0.844 (0.386-1.000)	0.805 (0.109-1.000)
**S**	0.050 (0.000-0.427)	0.046 (0.000-0.423)	0.053 (0.000-0.470)	0.071 (0.001-0.510)	0.032 (0.000-0.367)	0.036 (0.000-0.377)	0.044 (0.000-0.452)	0.021 (0.000-0.204)	0.042 (0.000-0.412)
**dC**	0.176 (0.000-0.618)	0.176 (0.000-0.618)	0.147 (0.000-0.618)	0.147 (0.000-0.500)	0.206 (0.000-0.647)	0.147 (0.000-0.706)	0.176 (0.000-0.735)	0.235 (0.000-0.588)	0.176 (0.000-0.647)
**Cw**	0.102 (0.000-0.330)	0.100 (0.000-0.320)	0.100 (0.000-0.330)	0.111 (0.000-0.337)	0.088 (0.000-0.334)	0.092 (0.000-0.330)	0.103 (0.000-0.360)	0.075 (0.000-0.286)	0.096 (0.000-0.330)
